# Type 2 Diabetes Mellitus in Latinx Populations in the United States: A Culturally Relevant Literature Review

**DOI:** 10.7759/cureus.23173

**Published:** 2022-03-15

**Authors:** Therese M Vidal, Caitlin A Williams, Uma D Ramoutar, Farzanna Haffizulla

**Affiliations:** 1 Internal Medicine, Nova Southeastern University Dr. Kiran C. Patel College of Allopathic Medicine, Fort Lauderdale, USA; 2 Internal Medicine, Nova Southeastern University Dr. Kiran C. Patel College of Osteopathic Medicine, Fort Lauderdale, USA

**Keywords:** immigrant health, health care disparity, diabetes mellitus management, socioeconomic factors, barriers to care, diet, cultural considerations, diabetes mellitus type 2, latinx health

## Abstract

Type 2 diabetes mellitus (T2DM) affects a large number of the American population. When compared to their representation in the general American population, a disproportionate number of Latinx individuals are affected. Within the Latinx American population, T2DM prevalence rates vary among individuals based on their country of origin. Deaths from T2DM among Latinx American population are also more compared to other ethnicities. This disparity underlines the importance of understanding the cultural considerations of T2DM disease presentation and management in Latinx communities, including risk factors, socioeconomic variables, and other social determinants of health such as access to care. There are various modifiable and non-modifiable risk factors for the development of T2DM, regardless of race. Staple foods in the diet of Latinx American communities, such as tortillas, rice, and beans, can cause spikes in blood sugar levels and can lead to obesity, which predisposes patients to develop T2DM. Latinx American populations suffer from lower access to healthcare than the general population due to many reasons, including language proficiency, immigration status, socioeconomic status, and level of acculturation. This study utilized the format of a commentary, while incorporating elements of a scoping review for data collection, to further explore these disparities and their impact on these populations. Understanding the cultural beliefs of Latinx individuals and how these beliefs contribute to the perceived development of T2DM is essential to properly treat these unique populations. Despite high rates of T2DM affecting Latinx individuals, non-adherence to prescribed diabetes medications is high among these populations. Interventions in the form of culturally tailored preventative education, in addition to active T2DM management, are necessary to combat the toll of this disease on Latinx Americans. Generic interventional techniques and methods should be replaced entirely by those that acknowledge, highlight, and utilize the sociocultural characteristics of Latinx Americans.

## Introduction and background

Type 2 diabetes mellitus (T2DM) affects 10.5% of Americans (34.2 million), with a disproportionate number being of Latinx or Hispanic descent [1]. The term “Latinx” is the “non-binary form of Latino or Latina,” meaning any individual with ancestry in Latin America [2]. Hispanic refers to someone from a Spanish-speaking country, which includes both Latin American countries and Spain [2]. When viewing age-adjusted prevalence among ethnic minorities, Latinx populations are ranked the second highest (12.5%) of all ethnicities [1]. Within the Latinx population in the United States, the prevalence among different ethnicities is as follows: Mexicans (14.4%), Puerto Ricans (12.4%), Central/South Americans (8.3%), and Cubans (6.5%) [1]. The disproportionate prevalence of diabetes in these Latinx communities within the United States is also demonstrated in their country of origin. For example, the prevalence of diabetes in Mexico is 13.5%, in Puerto Rico it is 13.7%, and in Cuba it is 9.6% [3]. Latinx Americans are known to have higher rates of uncontrolled T2DM, as indicated by higher hemoglobin A1c levels [4]. Poorly controlled T2DM is associated with worse outcomes, including subsequent cardiovascular disease, retinopathy, and chronic kidney disease (CKD) [4]. Deaths from T2DM in Latinx populations are also 1.25 times higher than non-Latinx populations [5]. Disparities experienced by Latinx Americans are apparent in the trends and statistics of disease prevalence among this community, for example, though T2DM is the major cause of CKD in Latinx individuals, those with CKD maintain poor management of T2DM, lack medication adherence, may be unaware of the association of CKD with T2DM, and have the potential to progress to ominous disease faster than non-Latinx communities [6,7]. The COVID-19 pandemic has further emphasized health disparities experienced by Latinx Americans, as these populations are experiencing higher rates of COVID-19 infection, potentially due to their increased likelihood of having a comorbid condition, such as T2DM [8]. These disparities underline the importance of understanding the cultural considerations of T2DM in Latinx communities, including risk factors and access to care. This commentary with a modified scoping review aims to build off the existing “Caribbean Diaspora Healthy Nutrition Outreach Project (CDHNOP): A Qualitative and Quantitative Approach to Caribbean Health” [9] by further exploring the current data available on the Latinx community related to T2DM and its associated comorbidities. This manuscript is meant to provide a general overview of the literature available on these topics and discuss the need for a more inclusive, personalized, and comprehensive approach to improving the health of Latinx communities.

## Review

Methods

Protocol

This study is a scholarly literature review with elements of a scoping review. We intended to primarily conduct a commentary but decided to incorporate aspects of Arksey and O’Malley’s scoping review framework for data collection [10]. Specifically, we loosely included some of their designated stages, including identifying a research question, identifying relevant studies, study selection, and summarizing the collected data. This study design was selected partially due to the sparsity of available data in the field of underserved and underrepresented communities.

Identifying the Research Question

The first step in this commentary included determining the research questions that would be addressed in our scoping review. Our research question was: “What is known from the existing literature about Type 2 Diabetes in Latinx populations?” We intentionally chose a more ambiguous research question because we wanted to maintain a wide approach to generate a larger breadth of coverage, as suggested by Arksey and O’Malley.

Identifying the Relevant Studies

Our search strategy included searching specific keywords on PubMed and Google Scholar for each area of interest in our study. Search strings always included “type 2 diabetes” AND “hispanic” OR “latinx.” Depending on the topic of interest, additional search terms would be added to the above string. Examples of these search strings include: type 2 diabetes AND hispanic OR latinx AND genetics, type 2 diabetes AND hispanic OR latinx AND obesity, type 2 diabetes AND hispanic OR latinx AND physical activity, type 2 diabetes AND hispanic OR latinx AND barriers to healthcare, and so on. These searches were conducted for each area of interest in our study, including genetics, obesity, cardiovascular disease, retinopathy, CKD, diet, physical activity, barriers to healthcare, cultural beliefs, management, and acculturation.

Study Selection

Due to the ambiguity of our research questions and basic search strings, a large number of irrelevant studies were generated on our initial search. Three reviewers performed data extraction and appraisal independently while adhering to loosely set inclusion and exclusion criteria to maintain some consistency in decision-making. The inclusion criteria included articles with a focus on Latinx populations, Hispanics, type 2 diabetes, cultural beliefs, diet, management, or comorbid conditions and sequelae of type 2 diabetes, including obesity, cardiovascular disease, hyperlipidemia, retinopathy, and CKD. Exclusion criteria included articles published before 2001. The decision to exclude articles was discussed among reviewers, and these articles were discarded after unanimous agreement. Some reasons for the exclusion of articles that may have otherwise met inclusion criteria include poor study design, lack of peer review, small sample size, study on the wrong population or focus on only one specific Latinx subgroup, or lack of significant findings.

Summarizing the Collected Data

Data collected from our literature review were directly used in the creation of our commentary piece. This commentary, which incorporated elements of the scoping review framework in the identification and selection of relevant articles, aimed to present a narrative account of the existing literature answering our primary research questions. The collected data were summarized in a paragraph format, organized by the area of focus (e.g., genetics, barriers to healthcare, etc.), and used to discuss the significance of culturally relevant care. Of note, scoping reviews do not aim to synthesize evidence or aggregate findings, as that is more the role of a systematic review.

Genetics of Latinx individuals contributing to T2DM

T2DM is a multifactorial disease with both modifiable and non-modifiable risk factors contributing to its development [11]. Though an emphasis is traditionally placed on environmental and modifiable risk factors, genetics also significantly contributes to the development of the disease as evidenced by greater rates of the disease in Latinx populations [11]. Genome-wide association studies (GWAS) have uncovered more than 100 genetic loci associated with the development of T2DM [12]; however, the accuracy of the resultant polygenic risk scores in the Latinx population is compromised by the fact that only 2% of the studied population is of Hispanic ancestry [11,12]. Few GWAS have been performed on Latinx populations in the United States, likely due to challenges in genetic mapping which may be attributable to the variability of their genome from the three main ancestries (American, European, and West African) [12]. Disruptions of SLC16A11 in Mexicans and Latin Americans have been associated with the development of T2DM due to altered fatty acid and lipid metabolism [12]. More recently, a GWAS of T2DM in the Latinx population in the United States identified two previously known association signals at the KCNQ1 locus [12]. Additionally, a novel single-nucleotide polymorphism (SNP) (SNP rs 1049549), likely an African ancestry-specific allele, was found to be consistent with T2DM across the Latinx population of the United States [13]. In accordance with a similar genetic risk score to European and Chinese populations, the Latinx population of the United States experiences a 7% increased risk of T2DM per associated allele [13].

Pathophysiological factors of T2DM in Latinx population

In addition to genetics, characteristics of the Latinx population that contribute to the development of T2DM include increased insulin resistance, compromised beta cell function and accelerated senescence, and an altered microbiome [10]. It has been suggested that the increased insulin resistance seen in the Latinx population is the result of higher obesity rates or genetic predisposition; it is likely due to a combinatorial effect [10]. One consequence of increased insulin resistance is a compensatory increased insulin secretion by pancreatic beta cells, which contributes to beta cell dysfunction and advanced senescence at a younger biological age than other ethnic groups [10]. As beta cell function ceases, the diagnosis of T2DM is made. Finally, the effect of an altered microbiome on the development of T2DM is not unique to the Latinx population; however, the reflection of the acculturated Latinx diet and antibiotic usage may be a unique explanation for the susceptibility of this population to the development of T2DM [10].

Comorbidities of T2DM in Latinx individuals

Several comorbidities associated with T2DM are seen at higher rates in Latinx populations, including obesity, cardiovascular equivalents, CKD, and retinopathy [14].

Obesity

Obesity, the presence of excess adipose tissue, is a well-known comorbid condition of T2DM and is one of the most important modifiable risk factors [14]. Due to the intertwining pathophysiology of obesity and T2DM, the term "diabesity" has been used to describe the coexistence of these diseases [15]. On a mechanistic basis, excess adipose causes adipocytes to hypertrophy and induces a configurational membrane change that interferes with the function of glucose transporters, resulting in increased insulin, or insulin resistance [16]. In turn, the impaired insulin resistance results in an increased amount of free fatty acids and the accumulation of excess adipose which, due to lipotoxicity of increased free fatty acids, contributes to heightened insulin resistance [17]. The most accepted screening tool for obesity, BMI, has been thoroughly evaluated in Hispanic populations. The Hispanic Community Health Study/Study of Latinos found a direct correlation between BMI and the prevalence of diabetes among Hispanic/Latinx populations [18]. Hispanic populations, both in the United States and their home countries, have higher rates of obesity than many other ethnic groups [19]. In 2017-2018, obesity in American Hispanics above 20 years was 44.8% prevalent, which is more than the non-Hispanic white and Asian populations and only less than the non-Hispanic black population [20]. In the younger population, Hispanics demonstrate the highest prevalence of youth obesity in the country, affecting 25.6% of this population [21]. Multiple explanations exist for the increased prevalence of obesity in Hispanics, the most influential of which may be sociocultural factors. In addition to diet and lack of exercise, the ideal body image in Hispanic populations has been described as “full-figured” due to the perceived connection with “wealth, affluence, and tranquility” [22].

Cardiovascular Equivalents

The excess adiposity seen in overweight and obese individuals is often concurrent with cardiovascular risk equivalents including hypertension and dyslipidemia and has therefore been suggested to play a prominent role in the development of both metabolic and cardiovascular diseases [23]. Molecular dysfunction secondary to obesity and diabetes induces vascular inflammation, resulting in vasoconstriction, thrombosis, and atherogenesis [24]. As such, Latinx populations are predisposed to the development of hypertension and hyperlipidemia due to their higher BMI and rates of obesity. In addition, Hispanic populations are more likely than any other race-ethnic group in the United States to have undiagnosed, undertreated, and uncontrolled hypertension [25]. Latinx individuals also have high rates of hyperlipidemia, a common comorbidity of T2DM [26,27]. Furthermore, physical activity is inversely associated with the development of both hypertension and hypercholesterolemia [28]. Latinx communities have been documented to have lower rates of physical activity than other ethnic groups in the United States [29].

Notably, the impact of cardiovascular disease on the Hispanic population has been an object of debate. The prevalence of other cardiovascular equivalents including abdominal aortic aneurysms, peripheral arterial disease, and carotid stenosis is lower in the American Hispanic population than in the white population [30]. It has been suggested that the prevalence and mortality rate of cardiovascular disease in the Hispanic population is less than that in non-Hispanic whites; however, the leading cause of death in those with T2DM was cardiovascular disease [31]. The Hispanic Paradox, which is described as a lower mortality rate despite the presence of multiple cardiovascular risk factors and comorbidities, is a perplexing phenomenon that may be explained by psychosocial factors and discrepancies in death certificate reporting; however, the exact reason for this phenomenon has yet to be elucidated [30].

Retinopathy

In addition to Latinx populations having higher rates of T2DM comorbidities, the incidence of T2DM complications, including diabetic nephropathy and retinopathy, is also increased. Though several mechanisms explain the development of retinopathy in the setting of T2DM, microvascular damage secondary to hyperglycemia or hypertension is a shared outcome [32]. The Los Angeles Latino Eye Study noted that the incidence of diabetic retinopathy among Latinx individuals was increased when compared with other races and ethnicities [33]. American Hispanics suffer from an increased rate of undetected eye diseases coupled with one of the highest prevalence rates of visual impairment in America [34]. Additionally, in those with self-reported T2DM, nearly 30% showed clinical signs of diabetic retinopathy [34]. It has been suggested that Latino populations are more reluctant to utilize eye care resources due to factors including the cost and lack of knowledge of preventative ocular health measures [34]. The high incidence of visual impairment, blindness, and worsening visual acuity and the relationship of progression of disease with age highlight the importance of targeted screening programs for older Latino populations [33].

CKD

CKD is defined as an altered state of kidney structure or function for more than three months and is most commonly attributable to diabetes and hypertension [35]. The pathophysiology of CKD secondary to T2DM is a complex interplay of various histopathological, hemodynamic, and metabolic, and inflammatory pathways that lead to chronic structural changes in the kidney that compromise integrity and function [36]. The Multi-Ethnic Study of Atherosclerosis found that compared to the white population, Hispanic populations had a higher incidence of CKD defined as a glomerular filtration rate less than 60 mL/min/1.73 m^2^ [37]. Without intervention, the progression of CKD to end-stage renal disease (ESRD) is nearly inevitable.

A study from Northern California showed that the incidence of ESRD is 1.5-fold higher in Hispanic populations when compared to non-Hispanic whites [38]. The progression of CKD has also been shown to be 81% greater among Hispanic populations compared to non-Hispanic whites when adjusted for sociodemographic and clinical characteristics, particularly in individuals with T2DM [37]. Specifically, American Dominicans and Puerto Ricans were shown to have a significantly faster decline in GFR compared to the white population [37]. Notably, even with using treatment strategies, Hispanics were less likely to achieve recommended management goals, indicating a likely progression of the disease, which is illustrated by the higher number of Hispanics receiving dialysis treatment than the white population [37].

Latinx diet as a factor in the development of T2DM

One of the most prominent risk factors for developing diabetes is a carbohydrate-rich diet, which is notable in many Latinx communities. Hispanic cuisine includes staples, such as tortillas, beans, and rice, especially among Puerto Rican, Dominican, and Mexican populations [39]. These foods cause spikes in blood sugar levels and can lead to obesity [39], which predisposes patients to develop T2DM [14]. Additionally, acculturation to the United States plays a role in the dietary patterns adopted by Latinx individuals. For example, it was found that less acculturated Latinx individuals were more likely to adhere to diets higher in fiber and lower in saturated fats [40], whereas more acculturated Latinx populations consume lower amounts of starchy roots, vegetables, and more fruits [41]. Food insecurity among newly immigrated Latinx populations could also potentially be attributed to their poor dietary habits. When analyzing the participants of the 2003-2010 National Health and Nutrition Examination Survey (NHANES), food insecurity was associated with a lower healthy eating index (HEI) among all ethnicities [42]. These communities were found to have an increased intake of added sugars and empty calories [42]. Although acculturated Latinx groups consume more fruits and low-starch vegetables, they are more likely to introduce processed foods and sweets into their diets [41]. When confronted with the potential of dietary restrictions for health purposes, Latinx patients with T2DM have expressed feeling restricted and uneasy [43]. Providing these populations with culturally tailored education on the importance of a healthier lifestyle and shaping these dietary recommendations to fit their cultural norms could potentially ameliorate the rates of T2DM. The Caribbean Diaspora Healthy Nutrition Outreach Project demonstrated that providing populations with culturally tailored nutrition education was effective at changing their food and beverage selection, specifically in Cuban and Dominican communities [9].

Physical inactivity among Latinx American populations

Among the ethnic subgroups in the United States, Latinx populations display the highest rates of physical inactivity. In a 2010 National Health Interview Survey, 45% of Latinx individuals stated that they never engaged in physical activity in their leisure time [44]. These higher rates of physical inactivity, even when adjusted for education levels, socioeconomic status (SES), employment, marital status, family income, and poverty, remain significant when compared to non-Hispanic whites [45]. As discussed previously, the level of physical activity in these populations can be inversely associated with an increased risk of developing some of the components and sequelae of metabolic syndrome, including hypertension, hypercholesterolemia, obesity, and cardiovascular disease [28]. Several factors have been cited as barriers to leisure-time physical activity in these subgroups. Health literacy, specifically knowledge about the benefits of exercise, and access to resources to engage in physical activity were noted as key factors in their ability to become physically active [45]. Other barriers include cultural perceptions of physical activity and pre-existing gender differences present in these societies [46]. For example, one study demonstrated that the two major reasons Latinas were less likely to be involved in physical activity included: (1) their belief that it would detract from their role as caregivers [47] and (2) their self-consciousness about their appearance. Interventions focused on providing education on the benefits of exercise as well as physical activity techniques that can be done without access to a standard gym could be useful in combating the physical inactivity reported in these populations [48].

Cultural-specific interventions, aimed at using their pre-existing belief system to motivate them to become more physically active, should also be considered. For example, Latinx culture places a strong emphasis on interpersonal relationships and family. Qualitative studies of these communities demonstrated social support as a significant motivator in whether or not Latinx individuals decided to pursue the physical activity in their leisure time [49-51]. Additionally, the Caribbean Diaspora Healthy Nutrition Outreach Project demonstrated a preference for walking, playing soccer, cricket, baseball, or going dancing as a form of exercise among Caribbean individuals [9]. They found that activities such as swimming and American football were unrelatable and unpopular forms of exercise for these communities [9]. With this knowledge, providers can work to make more culturally relevant exercise recommendations to their patients to improve various metabolic disorders prevalent among Latinx populations.

Barriers to healthcare experienced by Latinx American individuals

Latinx populations in the United States suffer from lower access to healthcare than the general population due to many contributing social factors, such as health literacy, language proficiency, immigration status, SES, and level of acculturation [52]. Health literacy, broadly defined as an individual’s ability to understand and navigate the healthcare system, has been shown to greatly contribute to health disparities [53]. Compared to other ethnicities, Latinx individuals in the United States have the lowest levels of formal education, including the highest rates of those who had not finished high school and the lowest rates of those who had achieved a bachelor’s degree or higher [54]. This may be because immigrants from those regions, in particular Mexico and Central America, have the lowest level of educational attainment than other countries of origin [55]. With regard to health literacy, ​​Latinx immigrants in the United States have lower levels of health literacy than other ethnicities [56]. Similarly, recent immigrants are more likely to be unfamiliar with the healthcare system, therefore serving as a barrier and delay to care [27]. In addition, having limited English proficiency not only restricts the care options available for Spanish-speaking patients, but further puts them at risk of misunderstanding their disease process and management plan [52]. This is of particular importance for diseases such as T2DM that require extensive active involvement from the patient, including lifestyle modifications, monitoring blood glucose, and proper medical management.

The lack of diversity in healthcare teams can also perpetuate inadequate access to healthcare services, as Latinx Americans are more likely to pursue treatment by Latinx physicians irrespective of their location and socioeconomic factors [52]. Their decision to choose physicians based on their cultural background and Spanish proficiency seems rooted in an inherent trust of Latinx providers, as these individuals believe that Latinx physicians can provide them with a higher quality of care solely based on their ethnicity [52,57].

SES, particularly health insurance status, is another barrier to care with Latinx individuals being more likely to be uninsured than non-Hispanic whites [52]. Specifically, nearly 20% of Latinx Americans are uninsured [58], with reports showing that uninsured Latinx Americans are less likely to seek medical care and treatment [59]. Undocumented immigrants have the added difficulty of not being eligible for certain federal benefits, including regular Medicaid [60,61]. Lack of insurance makes medical care less affordable due to greater out-of-pocket costs, putting additional financial strain on Latinx individuals from lower SES. This is significant when considering the high out-of-pocket costs of medications used to treat T2DM, including insulin, leading to nonadherence [62]. Additional SES barriers include limited transportation to healthcare appointments, lack of childcare during healthcare visits, and inability to take time away from work [52]. This is due to the lack of paid time off associated with many low-wage jobs [63], which Latinx individuals of lower SES tend to occupy [64].

Cultural components of management and treatment of T2DM

Perceptions of the self-management of diabetes among Latinx individuals contribute to the management of the disease. For example, a study that included predominantly Puerto Ricans in Massachusetts found that patients expressed difficulty controlling their diabetes, citing the time-intensive nature of monitoring the disease [65]. Furthermore, instead of turning to medical or social work services, these participants shared that they often turned to family or friends and then to their community or church, when they needed help with their health [65]. Similarly, a smaller study that focused on Mexican-Americans in the United States found that participants highlighted the familist aspect of diabetes care and management, with family members frequently monitoring their disease process [66]. Participants in this study also cited factors such as perceptions of the stigma of diabetes and lack of understanding of the disease process to be barriers to effective management [66].

While many Latinx individuals believe that biomedical factors, such as genetics, diet, and lack of exercise, predispose them to diabetes, many also believe that cultural beliefs and religious factors contribute to diabetes prevention and management in Latinx individuals, particularly those from lower SES [67,68]. Some Latinx populations believe that strong emotions can contribute to the development of diabetes. Specifically, susto, fear that is felt after a traumatic event, and coraje, emotions associated with social struggles, are viewed as causal factors [68]. Other Latinx individuals believe that developing diabetes is part of their fate, particularly rooted in religion, which is known as fatalismo [68]. Latinx adults have varying views on the development of diabetes, particularly when looking at the country of origin. For example, Latinx individuals from Mexico are more likely to attribute diabetes development to cultural beliefs, like those mentioned, while those from Puerto Rico are more likely to attribute diabetes development to religious belief, such as it being God’s will [67]. Thus, these differing viewpoints on the origin of diabetes make effective management more difficult, as some believe that nothing they could have done would have prevented the development of the disease, and others believe it can be effectively managed by controlling one's emotions and through prayer [67].

Cultural beliefs can often lead to the use of commercial and herbal products for the treatment of various medical conditions, including T2DM. Common herbal remedies for the treatment of T2DM among Latinx individuals include prickly pear cactus, aloe vera, celery, and chayote [69]. The efficacy of these herbal remedies has been shown, but with uncertain implications for clinical practice; for example, while prickly pear cactus has been shown to reduce serum glucose and insulin levels, likely due to its high fiber contents and hypoglycemic properties [70], aloe vera has shown to slightly improve glycemic control, but with great heterogeneity across studies [71], substances like celery have mostly shown promise for hyperglycemia control in rat models [72]. One study found that while nearly 70% of Latinx patients used herbal remedies, a majority reported that they did not disclose their use of herbal remedies to providers [69]. In another study, it was found that 84% of Mexican-Americans were aware of the use of herbs to treat medical conditions but more than one-third of these participants were not familiar with the specific herbs themselves or potential adverse effects associated with their use [57]. Additionally, Latinx individuals from Mexico, Puerto Rico, and the Dominican Republic were receptive to using standard and alternative treatment methods simultaneously, especially if the referring physician was fluent in Spanish [57]. These Latinx individuals reported that physicians who spoke Spanish were more credible sources [57]. However, a large observational study found that even after adjusting for the Spanish-language fluency of their physicians, Latinx individuals with limited English proficiency were less likely to be adherent to medication regimens, including both oral medications and insulin [73].

While insulin is often a mainstay of diabetes treatment for effective blood glucose control, many Latinx individuals have negative feelings toward the use of insulin. Latinx adults have been shown to believe that the use of insulin signals advanced diabetes and is associated with the onset of complications, including blindness and toe amputations [67]. Furthermore, Latinx individuals have expressed confusion about the timing of the onset of complications in relation to insulin use, as well as the safety of the drug due to feelings of dizziness, fatigue, palpitations, shakiness, and increased appetite after starting insulin [67]. Other options to treat T2DM also exist, including GLP-1 agonists like dulaglutide, which have shown to be efficacious in lowering HbA1c and weight in Latinx individuals with diabetes [74]. These findings highlight the importance of patient education about the development of type 2 diabetes and the options for treatment within Latinx communities.

Culturally tailored diabetes education intervention programs have shown to be successful for Latinx individuals. Many of these interventions focus on educating patients about self-management behaviors, including diet, physical activity, and self-monitoring of blood glucose levels, and monitoring their progress at adhering to these behaviors over time. One randomized control trial with mostly Puerto Ricans provided patients with either standard care or an intensive behavioral intervention, known as Latinos en Control, which provided a culturally tailored model over one year to address diabetes knowledge, attitudes toward diabetes care, and self-management behavior, while taking into consideration the health literacy of participants [75]. Session attendance was associated with greater reductions in HbA1c and improvement in dietary quality, including reductions in total calories and fat percentage [75]. A more recent randomized controlled trial with a larger sample size of Latinx patients in the United States provided less intensive intervention over six months in the form of integrated medical and behavioral visits with culturally tailored diabetes self-management education sessions. The results were similar in that participants taking part in the intervention had a greater reduction in HbA1c, total cholesterol, and diastolic blood pressure [76]. A smaller 3-month educational intervention program for type 2 diabetes tailored toward Mexican-Americans in Southern California showed an improvement in glycemic control and lipid profiles of participants with improved food choices and food monitoring [77].

Physicians can also become more culturally competent to provide more culturally tailored care. Specifically, one study investigated predictors of culturally competent care toward Mexican-American individuals. They found that physicians were more likely to have culturally relevant knowledge if they participated in diverse medical education settings and had experience in community clinics. Furthermore, providers who were of Latinx ethnicity and those who had bilingual skills were also more likely to be culturally aware [78]. This highlights the need for integrating teachings on the social determinants of health into undergraduate and graduate medical education.

Acculturation and its effects on the health of Latinx populations

Acculturation is defined as the cultural changes that take place when an individual adapts to the prevailing culture of a given society [79]. The effect to which Hispanic individuals acculturate to American society is multidimensional and dependent on a variety of factors, including the country of origin, age of entry into the United States, perceived ethnicity, ethnicity of an individual’s social circle, preference of language for media and entertainment, SES, educational level, sociocultural context, religious beliefs, family values, and health care practices [80]. Hispanic individuals that immigrate to cities that are densely populated with other Hispanic communities, such as Miami and New York City, are less likely to fully acculturate to American society if they choose to socialize only within these communities [81]. In Hispanic populations, it has been found that their healthcare practices and outcomes are associated with their level of acculturation [82]. It was found that higher rates of acculturation to American society was associated with increased levels of adherence to healthcare treatments and an increased propensity to use preventative healthcare [82]. Higher levels of acculturation are not always positive, as these individuals are also more likely to have high-fat diets and exhibit poorer eating habits [83]. The evolution of the cultural beliefs of these populations to that of the dominant culture in their community is highly variable but can provide explanations for some of their attitudes toward the healthcare system [84]. Understanding the role acculturation plays, while also considering the cultural beliefs and attitudes present in Latinx individuals, allows healthcare providers to cater their care to be more culturally competent and personalized.

## Conclusions

The prevalence of T2DM in the Latinx population in the United States is likely attributable to a number of key factors, most notably those related to the cultural and socioeconomic characteristics, including diet, language proficiency, immigration status, and cultural beliefs. The cruciality of addressing the disproportionate prevalence of T2DM in American Latinx communities is illustrated by the correspondingly heightened morbidity and mortality of this disease within the Latinx community when compared to other American immigrant subgroups. Thus, this study aimed to investigate the unique components that contribute to the development of type 2 diabetes in Latinx individuals, with a particular focus on culture, traditions, and beliefs. Interventions in the form of preventative education, in addition to active T2DM management, are necessary to combat the toll of this disease on Latinx Americans; however, generic interventional techniques and methods should be replaced entirely by those that acknowledge, highlight, and utilize the cultural and socioeconomic characteristics of the group.
